# A minor allele of the haplotype located in the 19q13 loci is associated with a decreased risk of hyper-LDL-cholesterolemia, and a balanced diet and high protein intake can reduce the risk

**DOI:** 10.1186/s12944-020-01352-1

**Published:** 2020-07-29

**Authors:** Sunmin Park, Suna Kang

**Affiliations:** Department of Food and Nutrition, Obesity/Diabetes Research Center, Hoseo University, 165 Sechul-Ri, BaeBang-Yup, Asan-Si, ChungNam-Do 31499 South Korea

**Keywords:** Dyslipidemia, Low-density lipoprotein, Apolipoprotein E, 19q13 loci, Protein

## Abstract

**Background:**

Although the human chromosome 19q13 loci are reported to be associated with hyper-LDL-cholesterolemia, the haplotype of single nucleotide polymorphism (SNP) has not been studied. Therefore, the association of the haplotype in 19q13 loci with hyper-LDL-cholesterolemia was determined and their interactions with lifestyles and nutrient intakes were evaluated in 28,445 Koreans aged > 40 years.

**Methods:**

SNPs were selected from 19q13 loci that had an association with hyper-LDL-cholesterolemia with the adjustment of confounders (age, gender, area of residence, and body mass index). Haplotype was constructed from the selected SNPs. An adjusted odds ratio of the haplotype for hyper-LDL-cholesterolemia and the interaction between haplotype and lifestyles was analyzed after adjusting for covariates.

**Results:**

Hyper-LDL-cholesterolemia had an association with apolipoprotein E (*APOE)*_ rs7259620, translocase of outer mitochondrial membrane 40(*TOMM40)*_rs157581, poliovirus receptor-related 2(*PVRL2)*_rs403155, exocyst complex component 3-like 2(*EXOC3L2)*_ rs10406604 and CD3e molecule-associated protein (*CD3EAP)*_rs3212986 in 19q13. The haplotype of these SNPs had a negative association with hyper-total-cholesterolemia and hyper-LDL-cholesterolemia by 0.669 and 0.684 times, respectively, after adjusting for covariates. The incidence of cardiovascular diseases, especially myocardial infarction, had a negative association with the minor alleles. The balanced diet pattern (BD) and protein intake had a significant interaction with the haplotype: the major-allele of the haplotype exhibited a positive association with hyper-LDL-cholesterolemia, compared to the minor allele, only when combined with a high intake of BD. The participants with the minor allele exhibited a lower hyper-LDL-cholesterolemia risk compared to those with the major allele only with high protein intake.

**Conclusion:**

The minor allele of haplotype located in 19q13 loci protected against hyper-LDL-cholesterolemia, especially with BD and high protein intake. The minor allele also had a negative association with myocardial infarction events.

## Introduction

The mortality from ischemic heart diseases has increased rapidly, possibly due to increased dyslipidemia [1]. The prevalence of hypercholesterolemia (≥240 mg/dL total cholesterol or taking cholesterol-lowering medication) in 1998, 2012, and 2016 has increased continuously from 7.2, 12.6, and 19%, and from 8.4, 14.9, and 20%, respectively, in Korean men and women aged ≥30 [1, 2]. Although the prevalence of dyslipidemia has increased, the awareness and treatment of dyslipidemia are relatively lower than for type 2 diabetes and hypertension [3]. In 2014, the Korean National Health Insurance changed the diagnostic criteria for dyslipidemia from a total cholesterol basis to a low-density lipoprotein cholesterol (LDL) basis because lowering the serum LDL concentration is the primary objective of dyslipidemia therapy [4]. In 2018, people with an LDL concentration of ≥160 mg/dL with one or more major risk factors were recommended to administer cholesterol-lowering medication and lifestyle modification [5]. The risk factors include middle-age (> 45 years for men and > 55 years for women), family history of premature coronary artery disease, hypertension, smoking, and hypo-high-density lipoprotein (HDL)-cholesterolemia. Elevated serum LDL concentrations need to be prevented by controlling the modifiable risk factors.

Although the genetic factors are unmodifiable, their effects can be modulated efficiently by environmental and lifestyle factors because genetic factors interact with lifestyle and environmental risk factors. Dietary patterns are one of the crucial factors for managing dyslipidemia, including hyper-LDL-cholesterolemia. The Mediterranean diet has been reported to be negatively associated with the serum LDL cholesterol concentrations in both randomized clinical trials and cross-sectional studies [6, 7]. Moreover, the Dietary Approaches to Stop Hypertension diet was reported to be negatively associated with the serum LDL concentrations [8]. Furthermore, a high-snack diet consisting largely of fried chips, cookies, sweets, ice cream, and liquid beverages is positively associated with the serum total cholesterol, LDL, and triglyceride concentrations [9]. A traditional healthy dietary pattern was reported to have an association with hyper- HDL-cholesterolemia concentrations in Korean men [10].

Cholesterol is taken up into the liver by chylomicrons and very-low-density lipoprotein (VLDL) remnants, where they are recognized by the apolipoprotein E (*APOE*) receptor [11]. When large amounts of cholesterol are stored in the liver due to a high cholesterol intake and synthesis, the LDL receptor (*LDLR*) is downregulated by lysosomal degradation after *LDLR* binds to the proprotein convertase subtilisin-kexin type 9 (*PCSK9*) [12]. The serum LDL concentrations are affected by *LDLR*, *PCSK9*, and *APOE* with the respective genes located in chromosomes 19, 1, and 19, respectively. Among the loci, 19q13 includes important genes related to cholesterol metabolism, including *LDLR*, *APOE*, *APOC1*, and translocase of outer-mitochondrial membrane 40 (*TOMM40*) [13, 14]. These genes are involved in LDL uptake into the cells to lower the serum LDL concentrations utilizing the triglyceride in VLDL to modulate the serum LDL concentrations. Furthermore, peroxisome proliferator-activated receptor-γ, which is involved in lipid utilization in the liver, is associated with the expression of the genes in the 19q13 loci [13]. In the Finnish cohort, the 19q13.31 and 19q13.32 loci exhibited genome-wide significance for serum LDL concentration, but the 1p32 loci did not [15]. On the other hand, the loci were reported to be associated with TG and HDL in non-Hispanic Caucasians and Africans [16]. These studies indicate that the genetic variants in 19q13.32 play an important role in the lipid metabolism, including hyper-LDL-cholesterolemia.

Dietary patterns do not uniformly affect dyslipidemia among all people because of the genetic impacts. No studies have investigated the interaction between genetic and environmental risk factors or how the results can be applied to people at risk of hyper-LDL-cholesterolemia for personalized nutrition. The current study aimed to determine if genetic variants located in 19q13 have a strong association with hyper-LDL-cholesterolemia and the incidence of cardiovascular diseases, including myocardial infarction and stroke. The interaction of haplotypes with lifestyles and nutrient intakes was also investigated to modulate hyper-LDL-cholesterolemia. These were examined in 28,445 Korean middle-aged and elderly participants in a large hospital-based urban cohort.

## Materials and methods

### Baseline characteristics of participants

In this study, 28,445 adults, aged 40 to 69 years, consisting of 10,261 men and 18,184 women, participated. The study procedures were compliant with the Declaration of Helsinki, and they followed the ethical standards of the responsible committee, including the Institutional Review Board of the Korean National Institute of Health for the KoGES (KBP-2015-055) and Hoseo University (1041231–150,811-HR-034-01). All participants provided written informed consent. The exclusion criteria in the participants were secondary obesity with Cushing’s disease, hypothyroidism, severely debilitating disease, undergoing anti-obesity treatment or weight loss for the past 6 months [17].

The baseline characteristics of the participants were collected from the survey, such as age, gender, education, income, and the status of smoking, drinking, and physical exercise. The daily physical activity was estimated by summing the multiplication of each activity level by the duration of physical activity at each activity level. The estimated daily physical activities were divided into low, medium, and high activity. The usual alcohol intake was determined by multiplying the average amount of alcohol consumed and the frequencies of alcohol drinking on each occasion. The daily alcohol consumption was divided into light drinking (< 1 g), moderate drinking (1-10 g), and heavy drinking (> 10 g). Daily coffee consumption was estimated in the same manner as the daily alcohol consumption. Non-smoking was defined as having smoked < 10 packs during their entire lifetime, while former smokers had not smoked for the last 6 months before the survey. A previous diagnosis of myocardial infarction and stroke events by a medical doctor was asked.

### Anthropometric and biochemical measurements

In the anthropometric measurements, the body weight, height, and waist and hip circumferences were measured three times as specified in a manual explained in previous studies [17, 18]. The body mass index was calculated by weight (kg)/height^2^ (m^2^) using the average values. In biochemical measurements of blood from the participants in an overnight-fasted state, the serum triglyceride, total cholesterol, and HDL concentrations, and plasma glucose concentrations were determined using a Hitachi 7600 Automatic Analyzer (Hitachi, Tokyo, Japan). The LDL was calculated using the Friedwald equation (total cholesterol – HDL – triglyceride/5 in the serum). Because Friedwald equation could not estimate serum LDL concentrations in serum triglyceride concentrations > 400 mg/dL [19], subjects with > 400 mg/dL were excluded.

### Nutrient intake assessment

The usual food intake of each participant was estimated using a semi-quantitative food frequency questionnaire (SQFFQ) that was validated by the three-day food-records for three seasons in a previous study [19]. The SQFFQ included 103 food items usually included in Korean meals. The portion sizes of each food item were categorized as half, one, and double of a single portion size to be shown as pictures. The amounts of each food item intake were measured by multiplying the frequencies of each food intake by its portion size [18]. The nutrient intakes, including energy, carbohydrate, protein, and fat, were calculated from the food intakes collected by the SQFFQ using Can-Pro 2.0 software designed for calculating the nutrient intake from the food intake. The nutrient program was developed by the Korean Nutrition Society (Seoul, Korea).

### Genotyping and quality control

Genotyping was conducted from the DNA extracted from the peripheral white blood cells of the subjects using the Axiom Biobank Plus Genotyping Array, KNIHv1.1 (Affymetrix, Santa Clara, CA, USA) [17]. Single nucleotide polymorphisms (SNPs) were excluded because they did not meet the criteria, including no gender bias, genotyping accuracy (> 98%), and low missing genotype call rate (< 4%) for quality control and quality assurance.

The genetic variants associated with the risk of hyper-LDL-cholesterolemia were examined through a genome-wide association study (GWAS), and 43 genetic variants were selected at chromosome 19 at *P* < 0.0001. According to Bayesian-Robust Linear Modeling of the Mahalanobis distance classifier genotype-calling algorithm, the discordance of batches with different batch sizes was approximately 2%. The gene names of the selected genetic variants were found using scandb.org, and SNPs related to LDL metabolism, including *APOE* in chromosome 19, were chosen. In the haplotype generation, the genetic variants included satisfied the following criteria: minor allele frequency (MAF) > 0.01, SNP missing rate < 0.1, and Hardy-Weinberg equilibrium (HWE; *P* > 0.05). Linkage disequilibrium (LD) analyses were performed in the selected genetic variants from the same chromosome using Haploview 4.2 in PLINK version 1.90b (http://pngu.mgh.harvard.edu/ ~purcell/plink). The SNPs with strong LD (D’ > 0.4) in the pairwise SNPs were excluded because they provided similar information to hyper-LDL-cholesterolemia risk.

### Genetic variants to have an association with hyper-LDL-cholesterolemia risk in Koreans

The participants were categorized into low-LDL and High-LDL groups by ≥160 mg/dL or taking cholesterol-lowering medication (case) and serum LDL concentrations < 160 mg/dL (control), regardless of age and gender based on the “Korean guidelines for the management of dyslipidemia in 2018” from the Korean Society of Lipid and Atherosclerosis and the Korean Association of Internal Medicine since the participants were > 40 years old. GWAS was conducted to explore the SNPs involved in hyper-LDL-cholesterolemia with the case and control groups with adjusting for the covariates of age, gender, residential area, and body mass index (BMI) using PLINK software. The gene names of the SNPs were identified using the SCAN database (http://scandb.org/newinterface/). The important SNPs that influenced the risk of hyper-LDL-cholesterolemia were selected, and their LD plot was generated using Haploview using PLINK version 1.90b software.

### Haplotype analysis

A haplotype is more powerful than a single SNP based analysis. SNPs adjacent to each other on the same chromosome were selected for haplotype analysis. Haplotype analysis was conducted from the SNPs selected from GWAS using PLINK software. Each SNP selected for the haplotype was given a score of 0, 1, or 2 according to the major allele, heterozygote allele, and minor allele, respectively. Haplotype scores were calculated by adding the scores of each SNP included in the haplotype. Haplotype alleles were categorized into three categories (0, 1–2, and 3–4) by tertiles for the major-allele (Major), heterozygote-allele (Heterozygote), and minor-allele (Minor) groups of the haplotype, respectively.

### Interactions between the haplotype and lifestyles on hyper-LDL-cholesterolemia

The daily nutrient intakes, including energy, protein, fat, and carbohydrate, as well as daily coffee and alcohol intake, were divided into high- and low-intake groups. The percentage of daily energy requirements consumed was calculated by dividing the daily energy intakes by the estimated dietary energy requirement of Koreans in 2016 [20]; 2400 kcal for < 50-year-old men; 2200 kcal for ≥50-year-old men; 1900 kcal for < 50 -year-old women; and 1800 kcal for ≥50-year-old women. The interactions between the lifestyles and haplotypes on hyper-LDL-cholesterolemia were analyzed using a two-way analysis of variance (ANOVA) including main effect terms and the interaction terms of haplotypes and lifestyles with adjusting for covariates. The covariates were the residential area, age, gender, BMI, alcohol and coffee intake, smoking status, menopause, and total activity.

### Statistical analysis

Statistical analysis was conducted using SAS version 9.4 (SAS Institutes, Cary, NC, USA). A two-sample t-test was used to analyze the statistical significance of the continuous variables, such as age, BMI, waist circumference, and glucose and lipid profiles, in the blood between the Low- and High-LDL groups. Their means and standard deviations were also calculated. The frequency distributions were analyzed in the classification variables (haplotype alleles, gender, myocardial infarction, and stroke) using a chi-squared test. Significant intergroup differences of the haplotype alleles were determined by one-way ANOVA by adjusting for different sets of covariates. Multiple comparisons of the groups were performed using a Bonferroni test.

The associations between the haplotype and the hyper-LDL-cholesterolemia risk were assessed using multiple logistic regression analysis, adjusting for different sets of covariates. The adjusted odds ratios (ORs) plus 95% confidence intervals (CI) were calculated using the major haplotype allele as a reference in the two models by multiple logistic regression analysis with an adjustment for two sets of covariates. The association of the genetic effect with hyper-LDL-cholesterolemia, according to the haplotype, was explored after offsetting all the potential effectors of hyper-LDL-cholesterolemia as covariates. The covariates of model 1 were the basic confounders, and those of model 2 included all potential confounders to influence the serum LDL concentrations. The covariates in model 1 were age, gender, residential area, BMI and energy intake, and the parameters in the model 1 plus energy, coffee, and alcohol intakes, smoking status, physical activity, and serum total cholesterol concentrations belonged to model 2 as covariates.

The interactions between the SNPs and lifestyles were determined by allocating the participants to low- and high-intake groups by the 75th percentiles. The interactions between the haplotype and lifestyles were analyzed by two-way ANOVA including an interaction term with covariates. Their effects on the hyper-LDL-cholesterolemia risk were assessed by multiple logistic analysis after adjusting for the covariates in the low- and high-groups. *P* values < 0.05 were considered significant.

## Results

### General characteristics of the participants according to hyper-LDL-cholesterolemia

The participants were older in the High-LDL group than the Low-LDL group in both genders. Subjects in the High-LDL group (≥160 mg/dL serum LDL cholesterol levels) were more likely to be obese (higher BMI and waist circumferences) than those in the Low-LDL group (< 160 mg/dL serum LDL cholesterol levels) in both genders (Table 1). Serum total cholesterol, HDL, LDL, and triglyceride concentrations in the High-LDL group were much higher than the Low-LDL group in both genders (Table 1). Therefore, both genders were combined for searching genetic variants to influence serum LDL concentrations. However, the incidence of cardiovascular diseases combined myocardial infarction and stroke was significantly higher in the High-LDL group than the Low-LDL group in women but not in men (Table 1). However, each event of myocardial infarction and stroke did not show significant differences between the Low-LDL and High-HDL groups in both genders. In the High-LDL group, cardiovascular disease incidence was higher by about 2.5 times than the Low-LDL group. When the exploration of genetic variants is conducted for cardiovascular diseases, gender differences need to be considered.

**Table 1:** General characteristics of the study population according to serum LDL concentration

	Men (*n* = 10,086)		Women (*n* = 18,086)	
	Low-LDL^1^ (*n* = 9073)	High-LDL^2^ (*n* = 1013)	Low-LDL^1^ (*n* = 15,336)	High-LDL^2^ (*n* = 2750)
Age (years)^3^	54.9 ± 8.4	55.8 ± 8.1^**^	52.5 ± 7.7	56.0 ± 6.7^***^
Body mass index (kg/m^2^)^3^	24.3 ± 2.7	25.0 ± 2.6^***^	23.6 ± 2.9	24.1 ± 2.9^***^
Waist circumference (cm)^3^	85.4 ± 7.5	87.3 ± 7.1^***^	78.0 ± 8.2	79.3 ± 7.8^***^
Hip circumference (cm)^3^	96.0 ± 5.5	96.0 ± 5.5	93.6 ± 5.6	93.1 ± 5.6^***^
Serum total cholesterol (mg/dL)^3^	187 ± 29.8	231 ± 44^***^	192 ± 25	240 ± 40^***^
Serum HDL cholesterol (mg/dL)^3^	48.8 ± 11.7	49.4 ± 10.8	54.9 ± 13	55.5 ± 39^*^
Serum LDL cholesterol (mg/dL)^3^	108 ± 27	152 ± 44^***^	114 ± 24	161 ± 12^***^
Ratio of LDL and HDL^3^	2.19 ± 0.76	3.02 ± 1.14^***^	2.34 ± 0.67	3.20 ± 0.98^***^
Serum TG (mg/dL)^3^	148 ± 104	153 ± 95	113 ± 74	121 ± 65^***^
Plasma glucose (mg/dL)^3^	98.5 ± 22.2	100 ± 24^*^	91.7 ± 21.5	94.2 ± 20.5^***^
Cardiovascular disease^4^ (number, %)	523 (5.76)	67 (6.61)	443 (2.89)	93 (3.38)^*^
Myocardial infarction (%)	370 (4.01)	45 (4.44)	319 (2.08)	66 (2.40)
Stroke (%)	153 (1.68)	20 (1.97)	124 (0.81)	27 (0.98)

The values represent means ± standard deviations or number of the subjects (percentage of each group). ^1^ Low serum LDL cholesterol concentrations < 160 mg/dl; ^2^ High serum LDL cholesterol concentrations ≥160 mg/dl and/or taking lipid-lowering medicine;

Significantly different from Low-LDL group in men and women at ^*^P < 0.05; ^**^P < 0.01; ^***^P < 0.001

### Selection of the genetic variants associated with hyper-LDL-cholesterolemia risk

**Fig. 1:**
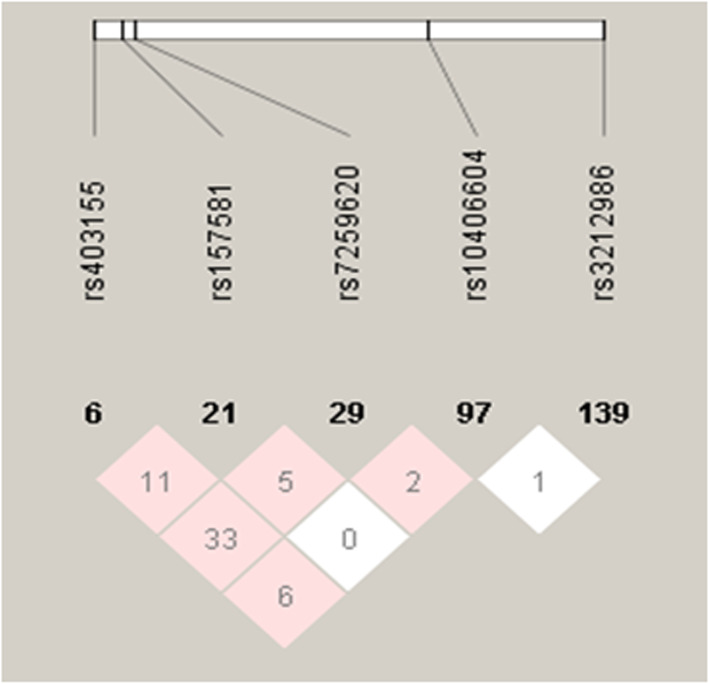
Haploview in the 19q13 loci. Haploview of the 19q13 loci. Each value in the colored rectangle represents the D’ between a pair of SNPs and the range of the value 0–100. The lighter color in the haploview indicates a lower correlation between the adjacent SNPs in the haplotype. The numbers in the triangles represent the correlation coefficient between the adjacent SNPs

In the 19q13 loci, hyper-LDL-cholesterolemia was associated with *APOE* rs7259620, poliovirus receptor-related 2 (*PVRL2*) rs403155 *TOMM40* rs157581, exocyst complex component 3-like 2 (*EXOC3L2*) rs10406604 and CD3e molecule associated protein (*CD3EAP*) rs3212986 and these SNPs were located from 45,363,299 to 45,912,736 in chromosome 19 (Table 2). Adjusted ORs of these SNPs were 0–1 and the *P* values were between 1.64E-4 and 3.79E-19 (Table 2). The SNPs satisfied the HWE criteria (*P* > 0.05). LD of 5 SNPs is presented in Fig. 1. The lighter color in the haploview indicated a lower correlation between the adjacent SNPs in the haplotype. The numbers in the triangles represented the D’ *100 between the adjacent SNPs [21]. Low correlations between adjacent SNPs (D’ < 0.2) suggested that the SNPs met the requirements to be included in the haplotype analysis. Although *EXOC3L2* rs10406604 and *CD3EAP* rs3212986 did not meet the GWAS statistical significance (*P* value < 5.0E-8), they showed statistical significance (1.94E-4 and 8.58E-05) and the haplotype including 2 SNPs provided a better model to show a more significant association with lipid profiles and cardiovascular diseases risk. The haplotype including 5 SNPs were used for further analysis. The logistic estimated coefficients and ORs of the haplotype including 3 SNPs were provided in Supplemental Table 1.

**Table 2:** The characteristics of the three genetic variants used for haplotype located in the 19q13 loci

SNP	Position	Mi	Ma	OR	P value for OR	MAF	HWE_P	Gene	Location
rs7259620	45,407,788	A	G	0.834	3.79E-19	0.284	0.737	*APOE*	near-gene-5
rs403155	45,363,299	T	C	0.789	3.30E-15	0.107	0.475	*PVRL2*	intron
rs157581	45,395,714	C	T	0.911	2.64E-19	0.236	0.250	*TOMM40*	coding-synonymous
rs10406604	45,723,986	A	G	0.917	8.58E-05	0.221	0.094	*EXOC3L2*	intron
rs3212986	45,912,736	A	C	0.926	1.64E-04	0.266	0.976	*CD3EAP*	missense

*APOE* Apolipoprotein E, *PVRL2* Poliovirus receptor-related 2 (nectin-2 and CD112), *TOMM40* Translocase of outer mitochondrial membrane 40 homolog, *EXOC3L2* Exocyst complex component 3 Like 2, *CD3EAP* CD3e molecule associated protein, *CHR* Chromosome, *SNP* Single nucleotide polymorphism, *Mi* Minor allele, *Ma* Major allele, *OR* Odds ratios (OR) for serum LDL concentration in the reference of the major allele; *P* value for OR adjusted for age, gender, residence area, body mass index, and energy intake, *MAF* Minor allele frequency, *HWE_P P* value for Hardy-Weinberg equilibrium

### Adjusted means of participant characteristics according to the haplotype generated from hyper-LDL-cholesterolemia

BMI and waist circumferences were not changed by haplotype but they were greater in participants in the High-LDL group than those in the Low-LDL group in two-way ANOVA (*P* < 0.001; Table 3). Serum total cholesterol, LDL, and triglyceride concentrations were lower in the carriers of the minor allele than those of the major allele (P < 0.001; Table 3) and they were higher in the High-LDL group in the than the Low-LDL group (P < 0.001). The ratio of LDL to HDL was influenced by the haplotype and LDL groups (P < 0.001) although serum HDL concentrations were not different according to LDL groups and haplotype (Table 3).

**Table 3:** Adjusted means in obesity status and serum lipid profiles of participants with major-, heterozygote-, and minor-alleles of the haplotype^1^ according to serum LDL cholesterol concentrations

	Low-LDL^2^			High-LDL^3^		
	Major^4^ (n = 10,950)	Heterozygote (n = 10,728)	Minor (*n* = 2731)	Major (*n* = 1782)	Heterozygote (*n* = 1680)	Minor (*n* = 301)
BMI (kg/m^2^)	23.8 ± 2.8^b^	23.9 ± 2.9^b^	23.9 ± 2.8^b^	24.3 ± 2.9^a^	24.4 ± 2.8^a^	24.2 ± 2.6^b+++^
WC (cm)	80.6 ± 8.7^b^	80.8 ± 8.8^b^	80.7 ± 8.6^b^	81.9 ± 8.3^a^	81.8 ± 8.2^a^	81.3 ± 7.4^b+++^
Total cholesterol (mg/dl)	191 ± 29^b^	190 ± 29^b^	186 ± 30^c^	238 ± 40^a^	239 ± 41^a^	235 ± 48^a***+++^
LDL (mg/dl)	114 ± 25^c^	112 ± 26^d^	106 ± 26^e^	159 ± 39^a^	158 ± 41^a^	153 ± 47^b***+++^
HDL (mg/dl)	52.6 ± 13	52.8 ± 13	53.3 ± 13	53.2 ± 12	53.1 ± 11	53.2 ± 12
TG (mg/dl)	123 ± 88^a^	125 ± 87^ab^	132 ± 91^c^	131 ± 77^bc^	136 ± 71^bc^	143 ± 83^c**+++^
Ratio of LDL/ HDL	2.34 ± 0.70^b^	2.31 ± 0.72^b^	2.22 ± 0.70^c^	2.65 ± 1.04^a^	2.62 ± 1.02^a^	2.56 ± 1.65^a***+++^

^1^Haplotypes of *APOE, PVRL2, TOMM40, EXOC3L2,* and *CD3EAP* in the 19q13 loci; ^2^ Low serum LDL cholesterol < 160 mg/dl and ^3^ High serum LDL cholesterol ≥160 mg/dl; ^4^Major, heterozygotes, and minor alleles of each SNP were scored 0, 1, and 2, respectively, and the scores of the SNPs in the haplotype were added. They were divided into 3 categories (Major, Heterozygote, and Minor haplotype groups) by 0, 1–2, and 3–4, respectively. Adjusted for age, gender, residence area, body mass index (BMI), and energy intake. Values represent adjusted means±standard deviations

Significantly different from major allele group at ^*^P < 0.05; ^**^P < 0.01; ^***^P < 0.001. Significantly different by LDL status ^+^*P* < 0.05; ^++^*P* < 0.01; ^+++^*P* < 0.001

^a,b,c^ Means without a common letter differ in the same row by Bonferroni test at *P* < 0.05

### A negative association of the haplotype associated with hyper-LDL-cholesterolemia

Adjusted ORs for hyper-total-cholesterolemia and hyper-LDL-cholesterolemia showed a negative association with the haplotype with the adjustment for model 1 covariates including BMI, residence area, gender, age, and energy intake and model 2 covariates including the parameters of model 1 plus smoking status, physical activity, menopause, serum total cholesterol levels and intake of energy, fat percent, carbohydrates percent, cholesterol, coffee, and alcohol (model 2; Table 4). Since there were no significant interactions with gender (*P* = 0.638), age (*P* = 0.120) and BMI (*P* = 0.989), the genetic effect on lipid profiles were analyzed in model 1 and 2 without separation by age, gender, or BMI.

**Table 4:** The estimated logistic regression coefficients (β’s), adjusted odds ratios and 95% confidence intervals for the risk of lipid profiles according to the alleles of the haplotype^a^ according to serum LDL cholesterol concentrations after covariate adjustments

	Model 1			Model 2	
	Major (n = 1782)	Heterozygote (n = 1680)	Minor (n = 301)	Heterozygote (*n* = 1680)	Minor (*n* = 301)
Total cholesterol^b^ (mg/dl)	1	0.084, 0.940 (0.893 ~ 0.988)	− 0.234^***^, 0.683 (0.629 ~ 0.742)	0.082, 0.928 (0.881 ~ 0.978)	−0.242^***^, 0.669 (0.614 ~ 0.730)
LDL^c^ (mg/dl)	1	0.098, 0.898 (0.853 ~ 0.946)	−0.249^***^, 0.675 (0.592 ~ 0.769)	0.089, 0.956 (0.842 ~ 0.975)	−0.237^***^, 0.684 (0.598 ~ 0.782)
HDL^d^ (mg/dl)	1	0.006, 0.969 (0.917 ~ 1.023)	−0.056, 0.908 (0.837 ~ 0.999)	0.031, 0.966 (0.912 ~ 1.023)	−0.110^***^, 0.845 (0.769 ~ 0.928)
Triglyceride^e^ (mg/dl)	1	−0.026, 1.088 (1.028 ~ 1.151)	0.125^***^, 1.243 (1.138 ~ 1.358)	−0.048, 1.110 (1.046 ~ 1.177)	0.188^***^, 1.387 (1.265 ~ 1.522)
Ratio of LD and HDL^f^	1	0.072^**^, 0.905 (0.854 ~ 0.959)	−0.244^***^, 0.667 (0.604 ~ 0.737)	0.041, 0.916 (0.876 ~ 1.000)	−0.160^***^, 0.770 (0.687 ~ 0.864)
Cardiovascular diseases	1	0.050, 1.018 (0.895–1.158)	−0.096, 0.858 (0.686–1.073)	0.076, 0.968 (0.846–1.108)	− 0.184^*^, 0.746 (0.592–0.941)
Myocardial infarction	1	0.067, 0.987 (0.783–1.245)	−0.121, 0.922 (0.624–1.363)	0.086, 0.965 (0.825–1.128)	−0.208^*^, 0.719 (0.549–0.943)
Stroke	1	0.002, 1.024 (0.882–1.189)	−0.033, 0.831 (0.639–1.082)	0.032, 0.961 (0.756–1.221)	− 0.105, 0.838 (0.559–1.256)

Values represent odds ratios and 95% confidence intervals

^a^Haplotypes of *APOE, PVRL2, TOMM40, EXOC3L2,* and *CD3EAP* in the 19q13 loci were generated by PLINK and it was divided into 3 categories (Major, Heterozygote and Minor haplotype groups) by the alleles (0, 1–2, and 3–4). The Major haplotype was the reference for both model 1 and model 2

The cutoff points for dividing the values of each parameter into 2 groups were as follows: the control group included < 200 mg/dL for serum total cholesterol concentrations^b^, < 160 mg/dL for serum LDL concentrations (LDL)^c^, ≥40 mg/dL for men and ≥ 50 mg/dL for women in serum HDL concentrations (HDL)^d^, < 150 mg/dL for serum triglyceride concentrations^e^ and 2.85 for the ratio of serum LDL concentrations to serum HDL concentrations^f^

Model 1: adjusted for age, gender, residence area, body mass index, and energy intake

Model 2: adjusted for the parameter in model 1 plus smoking, coffee, alcohol, physical activity, percent of fat and carbohydrate intakes, menopause, and serum total cholesterol concentrations

^*^ Significantly changed the risk of hyper-LDL-cholesterolemia by haplotype at *P* < 0.05, ^**^ at *P* < 0.01, and ^***^ at *P* < 0.0001

The adjusted ORs for hyper-total-cholesterolemia were lower in the carriers with minor alleles of the haplotype by 0.669 times in model 2 than in those with its major alleles (Table 4). Adjusted ORs for hyper-LDL-cholesterolemia in the carriers with minor alleles of the haplotype were lower by 0.675 and 0.684 times in models 1 and 2, respectively, compared to those with its major allele (Table 4). Moreover, hypo-HDL-cholesterolemia had a negative association with the haplotype in model 2, but not model 1, indicating that hypo-HDL-cholesterolemia risk was higher in the major allele of the haplotype, in comparison to its minor allele. The ratio of serum LDL/HDL was also significantly lower by 0.770 times in model 2, indicating that the participants carrying the minor allele of the haplotype exhibited lower ratios than those carrying the major allele (Table 4). However, serum triglyceride concentrations were the opposite of serum LDL concentrations. Participants with minor alleles had a hyper-triglyceridemia risk compared to those with the major allele.

Interestingly, the risk of cardiovascular diseases was lowered by 0.746 times in the minor allele of the haplotype than its major allele only in model 2 (Table 4). Among cardiovascular diseases, the events of myocardial infarction, but not a stroke, had a negative association with haplotypes in model 2.

### Interaction of haplotype and lifestyle in hyper-LDL-cholesterolemia

**Fig. 2:**
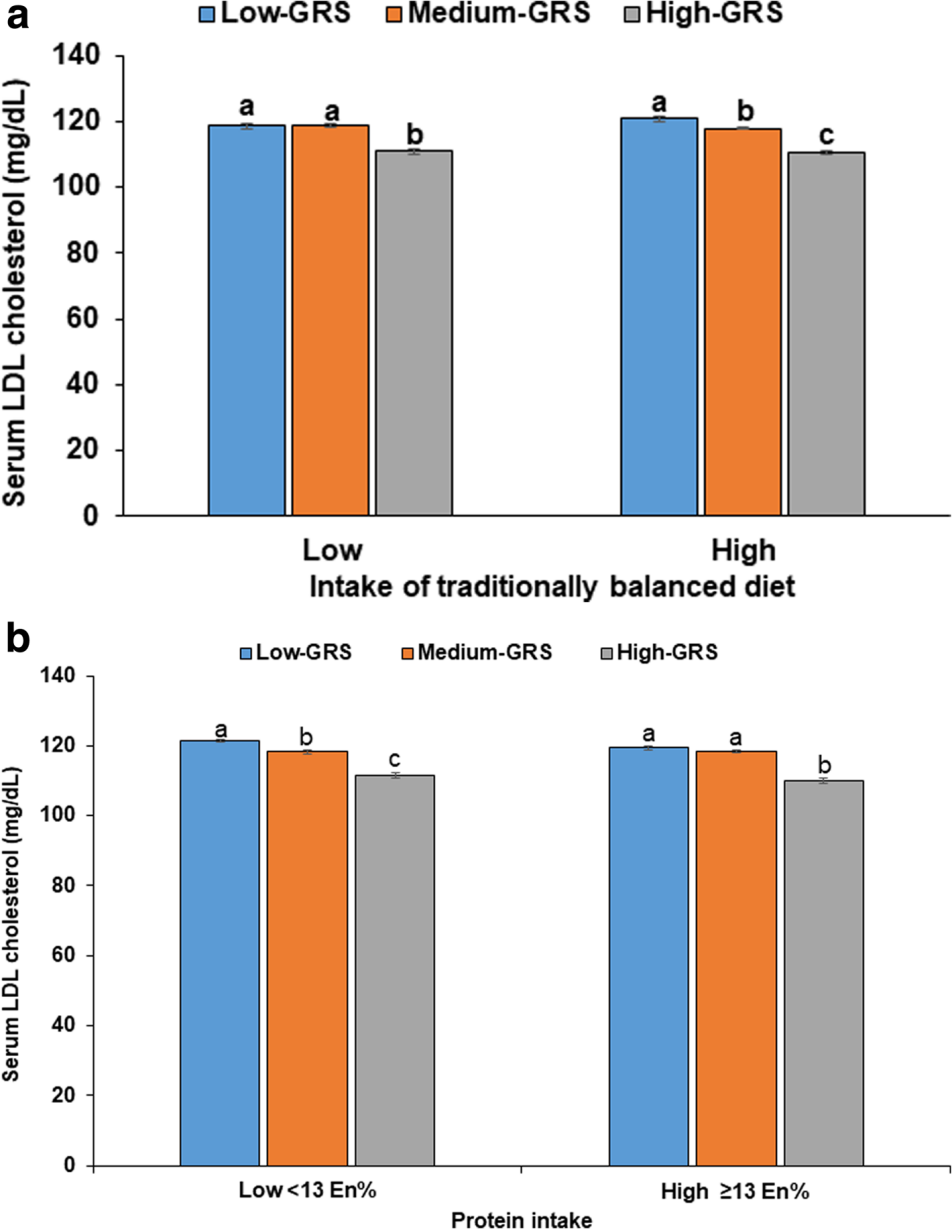
Serum LDL concentrations of participants with major, heterozygotes and minor alleles of haplotype with SNPs in the 19q13 loci. A: Balanced diet pattern (cutoff value: 75th percentiles); B: Protein intake (cutoff value: 13 energy percent (En%). Non-risk, heterozygotes, and risk alleles of each SNP were scored 0, 1, and 2, respectively, and the scores of each SNP in the haplotype were combined. The scores were divided into 3 categories (Major, Heterozygote, and Minor groups) by 0, 1–2, and 3–4, respectively. ^a,b,c^ Means without a common letter differ among the different allele groups by Bonferroni test at *P* < 0.05 in the low intake category. ^a’,b’,c’^ Means without a common letter differ among the different allele groups by Bonferroni test at *P* < 0.05 in the high intake category

A balanced diet pattern (BD) explained 44.4% of Korean diet patterns. The BD pattern had a positive association with consuming beans, potatoes, kimchi, green and white vegetables, mushrooms, fatty and white fish, seaweeds, fruits, and pickles (loading ≥0.4). A high intake of the BD pattern had an interaction with haplotype (*P* = 0.025). With low BD intake (the first tertile), hyper-LDL-cholesterolemia risk was higher in the major allele and the heterozygote allele than minor allele whereas the concentrations decreased in the descending order of major, heterozygote, and minor alleles, in the participants with high BD intake (Fig. 2a). The Heterozygote group had higher serum LDL concentrations with low BD intake (118.0 ± 31.1 mg/dL) than with the high BD intake (116.1 ± 30.2 mg/dL; Fig. 2a). Participants with the Western diet pattern had diets high in bread, cake, cookies, and fast food, which explained 23.2% of the total variance of the participants, and those in the rice-based diet pattern consumed mainly rice to explain 18.0% (Table 5). The Western diet pattern and mainly-rice diet pattern did not have interactions with haplotype and with a low or high intake of the Western diet and mainly-rice diet patterns, hyper-LDL-cholesterolemia risk had a similar association with haplotype (Table 5).

**Table 5:** Adjusted odds ratio for the risk of low serum LDL concentrations by the haplotype^a^ after covariate adjustments according to the patterns of lifestyles

	Major (*n* = 1782)	Heterozygote (n = 1680)	Minor (*n* = 301)	Gene-nutrient interaction *P* value
Low balanced diet^b^	1	0.994 (0.869–1.136)	0.722 (0.570–0.914)	0.0246
High balanced diet		0.854 (0.769–0.948)	0.640 (0.540–0.759)	
Low flour-rich diet^c^	1	0.876 (0.795–0.965)	0.540 (0.456–0.639)	0.6929
High flour-rich diet		0.881 (0.825–0.940)	0.596 (0.534–0.666)	
Low mainly-rice diet^d^	1	0.930 (0.845–1.023)	0.622 (0.528–0.732)	0.1788
High mainly-rice diet		0.858 (0.804–0.917)	0.560 (0.501–0.626)	
Low energy^e^	1	0.917 (0.861 ~ 0.977)	0.687 (0.586 ~ 0.805)	0.2870
High energy		0.918 (0.799 ~ 1.056)	0.680 (0.529 ~ 0.873)	
Low carbohydrate^f^	1	0.898 (0.848 ~ 0.952)	0.678 (0.585 ~ 0.785)	0.1922
High carbohydrate		0.931 (0.765 ~ 1.134)	0.718 (0.515 ~ 1.002)	
Low protein^g^	1	0.927 (0.834 ~ 1.031)	0.694 (0.576 ~ 0.836)	0.0059
High protein		0.933 (0.870 ~ 0.999)	0.671 (0.544 ~ 0.815)	
Low fat^h^	1	0.876 (0.713 ~ 0.993)	0.637 (0.527 ~ 0.770)	0.1690
High fat		1.034 (0.929 ~ 1.151)	0.734 (0.606 ~ 0.888)	
Low alcohol drinking^i^	1	0.898 (0.851 ~ 0.947)	0.645 (0.554 ~ 0.751)	0.3690
High alcohol drinking		0.910 (0.765 ~ 1.084)	0.868 (0.649 ~ 1.161)	
Non- + former smoking	1	0.910 (0.862–0.962)	0.697 (0.608–0.800)	0.3436
Smoking		0.841 (0.717–0.987)	0.579 (0.440–0.762)	
Low coffee intake^j^	1	0.933 (0.864 ~ 0.948)	0.679 (0.589 ~ 0.782)	0.0935
High coffee intake		1.139 (0.890 ~ 1.457)	0.762 (0.504 ~ 1.151)	
Low physical activity^k^	1	0.876 (0.812 ~ 0.944)	0.609 (0.504 ~ 0.735)	0.1105
High physical activity		0.887 (0.698–1.127)	0.738 (0.610 ~ 0.970)	

^a^Haplotypes of *APOE, PVRL2, TOMM40, EXOC3L2,* and *CD3EAP* were generated by plink and they were divided into 3 categories (Major, Heterozygote and Minor haplotype groups) by the alleles (0, 1–2, and 3–4). Reference was the major haplotype allele

Values represent odds ratios and 95% confidence intervals

The cutoff points for dividing the values of each parameter into 2 groups were as follows: less than 75th percentile of the respective diet intake^b,c.d^, less than estimated energy intake^e^, less than 13% protein^f^, 70% carbohydrate (CHO)^g^, 15% fat^h^, 20 g alcohol per day^i^, less than 10 cups of coffee per week^j^, and 1 h moderate activity per day^k^. Multivariate regression models include the corresponding main effects, interaction terms of gene and main effects (energy and nutrient intake), and potential confounders such as BMI, residence area, gender, age, smoking, coffee, alcohol, physical activity and serum total cholesterol concentrations

Energy, carbohydrate, and fat intakes did not have interactions with the haplotype to influence hyper-LDL-cholesterolemia. However, protein intakes had an interaction with the haplotype (*P* = 0.006). The serum LDL concentrations in the Major group with high protein intake (119.4 ± 31.9 mg/dL) were lower than those in participants with low protein intake (121.4 ± 32.1 mg/dL; Fig. 2b). Participants with the minor allele (111.3 ± 32.2 mg/dL) had much lower LDL-cholesterol concentrations than those with the major allele (120.7 ± 32.0 mg/dL).

## Discussion

The 19q13 loci are important for modulating serum total-cholesterol and LDL concentrations in people of various ethnicities with family histories of type 2 diabetes [22]. In the current study, the haplotype comprised of 5 genetic variants in 19q13 had a strong association with hyper-LDL-cholesterolemia risk after adjusting for covariates. Hyper-LDL-cholesterolemia was associated with *APOE* rs7259620, *PVRL2* rs403155, *TOMM40* rs157581, *EXOC3L2* rs10406604 and *CD3EAP* rs3212986 in the 19q13 loci. The haplotype included 5 selected SNPs that are associated with VLDL remnant delivery into the liver, conversion from VLDL to LDL in the blood, and LDL release into the circulation. Hyper-LDL-cholesterolemia risk was lower in the carriers of the minor alleles of the haplotype compared to carriers of the major allele, especially in those who had a high intake of the balanced diet pattern. The carriers with the minor allele had lower hyper-LDL-cholesterolemia risk compared to those with the major allele, especially in the high protein intake group. These results suggested that a personalized nutrition approach to the modulation of dietary patterns and nutrient intakes can reduce the risk of cardiovascular diseases and hyper-LDL-cholesterolemia. This is the first study to show an interactive association between haplotype and nutrition intake for modulating LDL-cholesterol and cardiovascular disease risk.

LDL metabolism is closely associated with VLDL metabolism [11]. Thus, cholesterol and triglyceride in the circulation are interrelated and genetic factors may interact with dietary intake. The present study showed that participants having high serum LDL concentrations also had a high concentration of not only total cholesterol and triglyceride but also HDL. Since the increase of serum HDL concentrations were small, the ratio of LDL to HDL in the serum was much higher in the High-LDL than the Low-LDL. Furthermore, the incidence of cardiovascular diseases was much higher in the High-LDL than the Low-LDL even with the cutoff of 160 mg/dL serum LDL concentrations [23]. Thus, serum LDL concentrations need to be controlled with the modifications of lifestyles according to personal genetic variants.

Genetic variants that regulate serum LDL levels are reported to vary among ethnicities. *LDLR* rs6511720 and rs57217136 are involved in enhancer-binding protein sites for a transcription factor for *LDLR* and participants in this study who carried their minor alleles had increased *LDLR* expression, which consequently reduces serum LDL concentrations [24]. G carriers of *PCSK9* rs562556 had a lower risk of dyslipidemia including decreased serum LDL concentrations in meta-analysis without separating ethnicities [25]. Participants with the combination of *ATP-binding cassette sub-family G member-5* rs6720173-C, *cholesterol 7 alpha-hydroxylase* rs3808607-TT, and *7-dehydrocholesterol reductase* rs760241-GG genotypes had lower serum LDL concentrations relative to those with opposite genotypes after a blended dairy consumption for 4 weeks (3 servings/day) [26]. The *APOE-ε4* variant elevates serum LDL and homocysteine concentrations, thereby increasing the risk of cardiovascular diseases. Participants with CT and TT at *methylenetetrahydrofolate reductase* C667T are at higher risk of cardiovascular diseases than those with the CC genotype [27]. In the present study, genes in the 19q3 loci modulated serum LDL concentrations. The SNPs were *APOE* rs7259620, *PVRL2* rs403155, *TOMM40* rs157581, *EXOC3L2* rs10406604, and *CD3EAP* rs3212986. In Han Chinese (784 with cardiovascular diseases and 730 without cardiovascular diseases) *APOE* rs7259620 had a positive association with cardiovascular diseases only in men, not women without adjusting for the covariates [28]. The study explained that the association may be due to gender differences in hormonal profiles, smoking status, alcohol-drinking, occupation, and dietary habits. These results are different from the present study in which *APOE* rs7259620 had a negative association with serum LDL concentration and cardiovascular disease incidence. Participants with minor allele had lower serum LDL concentrations and cardiovascular disease risk than those with major allele.

PVRL2 is an adherens junction protein. It responds to plasma cholesterol concentrations and it is involved in transendothelial migration of leukocytes to modulate vascular inflammation in *PVRL2* deficient mice and presumably acts similarly in humans. Thus, PVRL2 is associated with the development of atherosclerosis in the endothelial sites [29]. TOMM40 is a protein embedded into the mitochondria outer membranes and is required for the movement of proteins into mitochondria. *TOMM40* and *APOE-TOMM40* loci are associated with dyslipidemia in Asians and non-Asians [30, 31]. *EXOC3L2* is shown to interact with *EXOC4* is a component of the exocyst complex. The complex is involved in exocytic vesicles of the cell membrane which mediate the process of exocytosis. It may be associated with LDLR exocytosis. It also regulates cell polarity and cell migration in endothelial cells. *EXOC3L2* has not been previously reported to modulate dyslipidemia but *EXOC3L2, APOE, TOMM40,* and *PVRL2* are reported to be Alzheimer’s disease-susceptibility genes [32, 33]. *CD3EAP* encodes a nucleoprotein that is localized to the fibrillar centers of the nucleolus and it is mainly reported to be involved in lung cancer [34]. However, no previous studies have shown it to be related to dyslipidemia and cardiovascular diseases. Therefore, it was novel to demonstrate that the haplotype of 19q3 loci was shown to be related to the modulation of serum LDL concentrations in this study. The major alleles of the haplotype were a genetic risk for hyper-LDL-cholesterolemia.

Typically, circulating concentrations of LDL and triglyceride increase and decrease in parallel [35]. An unusual feature of these data was that the minor allele carriers at low risk of hypercholesterolemia were at elevated risk of hypertriglyceridemia. This may be due in part to the alleles decreasing biosynthesis of cholesterol in the liver while not affecting the availability of triglycerides derived from the diet. This would have the effect of increasing the content of triglyceride in VLDL and LDL particles relative to cholesterol. Genetic alterations that result in elevated triglycerides often involve variants that lower the activity of lipoprotein lipase, thereby impairing the removal of triglyceride from lipoprotein particles [35]. It may be possible that some of the genetic variants also affected triglyceride concentrations in such a manner. However, that cannot be determined from the current research data.

Overweight and obesity are risk factors for dyslipidemia [36]. Dietary components are also reported to modulate serum LDL concentrations [37]. The present study showed that energy, carbohydrates, fat intake did not have an interaction with the haplotype to modulate serum LDL concentrations. However, protein intake had an interaction with the haplotype. Carriers with minor alleles had lower hyper-LDL-cholesterolemia risk compared to those with major allele when consuming high amounts of foods in the balanced diet pattern as well as protein. However, the flour-rich diet and mainly-rice diet patterns did not have interactions with the haplotype. Other nutrients, coffee, and alcohol intake did not interact with haplotype to affect hyper-LDL-cholesterolemia risk.

### Strengths and limitations

This study was the first study to investigate the haplotype in 19q13 loci that were associated with hyper-LDL-cholesterolemia risk and the haplotype interacted with a balanced diet pattern and protein intake. In the haplotype of 19q13 loci, the major allele was the risk allele and a higher proportion of people had a genetic risk to have hyper-LDL-cholesterolemia, Genetic differences were associated with the events of cardiovascular diseases, especially myocardial infarction. These results can be used for the prevention of not only hyper-LDL-cholesterolemia but also cardiovascular disease events. The balanced diet pattern and high protein intake protected against hyper-LDL-cholesterolemia in Major haplotype.

However, there were some limitations. 1) The data of this current study came from a cross-sectional setting even from a large hospital urban cohort. The results did not demonstrate cause and effect relationships. 2) Two SNPs included in the haplotype (rs10406604 and rs3212986) did not meet the conservative statistical significance (*P* < 5.0E-8). These SNPs might not be significantly associated with hyper-LDL-cholesterolemia. 3) Food intake was calculated from SQFFQ that was validated by 3-day food records [38]. The results might be somewhat under- and over-estimated for usual food intake. The fat intake was estimated but saturated, mono-unsaturated, and polyunsaturated fatty acid intake were not provided in KOGES although their fatty acid intake associated with hyper-LDL-cholesterolemia.

## Conclusion

The present study suggested that the participants with hyper-LDL-cholesterolemia (≥160 mg/dL) were at higher risk of myocardial infarction and cardiovascular disease. Participants carrying the major allele haplotype in 19q13 loci had a higher risk of hyper-LDL-cholesterolemia by 1.5 times, compared to those carrying the major allele. The incidence of cardiovascular diseases, especially myocardial infarction, also had a positive association with the major allele of the haplotype. Participants carrying the major allele of the haplotype of 19q13 loci could reduce hyper-LDL-cholesterolemia and cardiovascular risk by consuming a balanced dietary pattern and with high protein intakes. Westernization of diet patterns can increase the susceptibility of hyper-LDL-cholesterolemia and cardiovascular diseases since the majority of people have risk alleles potentially in Asians including Korea. These results can be applied to precision nutrition protocols to prevent lowering serum LDL concentration and cardiovascular diseases and promote healthy aging.

## Supplementary information

**Additional file 1.** Supplemental Table 1 was provided in online only.

## Data Availability

The datasets used and/or analyzed during the current study are available from the corresponding author on reasonable request.

## Abbreviations

LDL: Low-density lipoprotein cholesterol
HDL: High-density lipoprotein cholesterol
*APOE*: Apolipoprotein E
*LDLR*: LDL receptor
*PCSK9*: Proprotein convertase subtilisin-kexin type 9
SQFFQ: Semi-quantitative food frequency questionnaire
GWAS: Genome-wide association study
HWE: Hardy-Weinberg equilibrium
MAF: Minor allele frequency
BMI: Body mass index
ANOVA: Analysis of variance
ORs: Odds ratios
CI: 95% confidence intervals
SNP: Single nucleotide polymorphism
*PVRL2*: Poliovirus receptor-related 2
*TOMM40*: Translocase of outer mitochondrial membrane 40
*EXOC3L2*: Exocyst complex component 3-like 2
*CD3EAP*: CD3e molecule associated protein
LD: Linkage disequilibrium
VLDL: Very-low-density lipoprotein
Major: The major allele of the haplotype
Heterozygote: The heterozygote allele of haplotype
Minor: The minor allele of the haplotype
BD: A balanced diet pattern
