# Supplementary material for: A minor allele of the haplotype located in the 19q13 loci is associated with a decreased risk of hyper-LDL-cholesterolemia, and a balanced diet and high protein intake can reduce the risk
Source: Lipids Health Dis. 2020 Jul 29;19:178. doi: 10.1186/s12944-020-01352-1 (PMC7391697; doi:10.1186/s12944-020-01352-1)
Supplement: Supplementary file 1 — Additional file 1. Supplemental Table 1 was provided in online only. [file 12944_2020_1352_MOESM1_ESM.docx]

Supplemental Table 1. The estimated logistic regression coefficients (β's), adjusted odds ratios and 95% confidence intervals for the risk of various lipid profiles according to the alleles of the haplotype^1^ according to serum LDL cholesterol concentrations after covariate adjustments

|  | Model 1 | | | Model 2 | |
| --- | --- | --- | --- | --- | --- |
|  | Major  (n=1,782) | Heterozygote  (n=1,680) | Minor  (n=301) | Heterozygote  (n=1,680) | Minor  (n=301) |
| Total cholesterol^2^  (mg/dl) | 1 | -0.026, 0.806 (0.729, 0.891) | -0.163^***^, 0.703 (0.640, 0.772) | -0.020, 0.810 (0.731, 0.899) | -0.171^***^, 0.697 (0.632, 0.767) |
| LDL^3^  (mg/dl) | 1 | -0.093^**^, 0.670 (0.568, 0.791) | -0.214^***^, 0.594 (0.508, 0.695) | -0.059, 0.769 (0.629, 0.939) | -0.145^***^, 0.705 (0.584, 0.852) |
| HDL^4^  (mg/dl) | 1 | -0.046, 0.900 (0.806, 1.002) | -0.016, 0.925 (0.835, 1.024) | -0.049, 0.860 (0.767, 0.965) | -0.053^*^, 0.856 (0.769, 0.953) |
| Triglyceride^5^  (mg/dl) | 1 | -0.018, 1.094 (0.981, 1.220) | 0.126^***^, 1.263 (1.141, 1.398) | 0.007, 1.202 (1.073, 1.347) | 0.170^***^, 1.202 (1.073, 1.347)^***^ |
| Ratio of LDL and HDL^6^ | 1 | -0.07, 0.742 (0.656, 0.838) | -0.158^***^, 0.679 (0.606, 0.761) | -0.041, 0.832 (0.723, 0.894) | -0.101^***^, 0.784 (0.687, 0.894) |
| Cardiovascular diseases | 1 | 0.018, 0.941 (0.719, 1.080) | -0.096, 0.840 (0.653, 1.080) | -0.032, 0.698 (0.535, 1.060) | -0.164^**^, 0.797 (0.535, 0.912) |
| Myocardial infarction | 1 | 0.016, 0.944 (0.690, 1.291) | -0.090, 0.848 (0.634, 1.135) | -0.035, 0.794 (0.570, 1.104) | -0.161^*^, 0.700 (0.514, 0.954) |
| Stroke | 1 | -0.030 0.842 (0.509, 1.394) | -0.112, 0.775 (0.483, 1.244) | -0.71, 0.740 (0.435, 1.257) | -0.159, 0.677 (0.411, 1.116) |

Values represent odds ratios and 95% confidence intervals.

^1^Haplotypes of *APOE,* *PVRL2,* and *TOMM40* in the 19q13 loci were generated by PLINK and were divided into 3 categories (Major, Heterozygote and Minor groups) by the alleles (0, 1-2, and 3-4). The Major haplotype was the reference for both model 1 and model 2.

The cutoff points for dividing the values of each parameter into 2 groups were as follows: the control group included < 200 mg/dL for serum total cholesterol concentrations^2^, <160 mg/dL for serum LDL concentrations (LDL)^3^, ≥40 mg/dL for men and ≥50 mg/dL for women for serum HDL concentrations (HDL)^4^, <150 mg/dL for serum triglyceride concentrations^5^ and 2.85 for the ratio of serum LDL concentrations to serum HDL concentrations^6^.

Model 1: adjusted for age, gender, residence area, body mass index, and energy intake.

Model 2: adjusted for the parameters in model 1 plus smoking, coffee, alcohol, physical activity, percent of fat and carbohydrate intakes, menopause, and serum total cholesterol concentrations.

^*^ Significantly changed the risk of hyper-LDL-cholesterolemia by haplotype at *P*<0.05, ^**^ at *P*<0.01, and ^***^ at *P*<0.0001.
